# Solvent Tolerance Improvement of Lipases Enhanced Their Applications: State of the Art

**DOI:** 10.3390/molecules29112444

**Published:** 2024-05-22

**Authors:** Mei Chen, Tongtong Jin, Binbin Nian, Wenjun Cheng

**Affiliations:** State Key Laboratory of Materials-Oriented Chemical Engineering, School of Pharmaceutical Sciences, Nanjing Tech University, Nanjing 210009, China; chenmei13548508118@163.com (M.C.); 202121009061@njtech.edu.cn (T.J.); bbnian@njtech.edu.cn (B.N.)

**Keywords:** solvent tolerance, organic solvents, ionic liquids, deep eutectic solvents, protein engineering

## Abstract

Lipases, crucial catalysts in biochemical synthesis, find extensive applications across industries such as food, medicine, and cosmetics. The efficiency of lipase-catalyzed reactions is significantly influenced by the choice of solvents. Polar organic solvents often result in a decrease, or even loss, of lipase activity. Conversely, nonpolar organic solvents induce excessive rigidity in lipases, thereby affecting their activity. While the advent of new solvents like ionic liquids and deep eutectic solvents has somewhat improved the activity and stability of lipases, it fails to address the fundamental issue of lipases’ poor solvent tolerance. Hence, the rational design of lipases for enhanced solvent tolerance can significantly boost their industrial performance. This review provides a comprehensive summary of the structural characteristics and properties of lipases in various solvent systems and emphasizes various strategies of protein engineering for non-aqueous media to improve lipases’ solvent tolerance. This study provides a theoretical foundation for further enhancing the solvent tolerance and industrial properties of lipases.

## 1. Introduction

Lipases (EC 3.1.1.3), known for their potent efficiency in diverse chemical reactions including hydrolysis, ammonolysis, acidolysis, alcoholysis, and transesterification, have extensive applications across various industries such as food, textiles, leather, cosmetics, papermaking, detergents, and biodiesel production [1] (Table 1). Compared to biocatalysis in the aqueous phase, non-aqueous phase biocatalysis not only promotes the solubilization of hydrophobic substrates and enhances the mass transfer effect during the reaction process, but also reduces the undesirable side reactions (e.g., hydrolysis, polymerization, etc.) in which water is involved, and thus improves the efficiency of the reaction [2]. For example, in the synthesis of SCH51048, a broad-spectrum orally active antifungal agent, an intermediate bearing two hydroxyl groups was selectively converted to a monoacetate in an anhydrous environment using Novozym 435 (an immobilized lipase) as a catalyst and acetonitrile as a solvent. This enzymatic process is maintained in operation in the pilot plant and is designed to provide a continuous supply of materials needed for clinical trials [3]. However, the drawbacks of solvent residues, volatility, and poor enzyme compatibility of the traditional organic solvents hinder their further applications in many enzymatic syntheses. Although some previous studies suggested that the hydrophobic organic solvents promoted the “interfacial activation” of lipases and thus were beneficial in maintaining the “open state” of the lid, their low solubility for polar substrates often leads to low enzymatic efficiency [4]. In addition, some amphiphilic and strongly polar solvents such as dimethyl sulfoxide (DMSO) can easily form a strong interaction with enzymes and thus can easily denature their structures, resulting in a loss of their activity [5]. Therefore, new green solvents such as ionic liquids (ILs) and deep eutectic solvents (DESs) have been gradually developed for various enzyme-catalyzed reactions. In addition, various strategies have been developed to enhance the solvent tolerance of lipases such as enzyme immobilization [6], screening of novel enzymes [7], and chemical modification [8]. Especially, protein engineering exhibited its great advantages, such as high efficiency and low cost, in improving the solvent tolerance of various enzymes [9]. However, contrary to the well-established strategies for thermal stability engineering, the general approach for engineering solvent tolerance is relatively underdeveloped, which may be because enzyme solvent tolerance is typically influenced by a multitude of factors including the solvents themselves, substrates, temperature, pH, and more.

In this review, the effects of different non-aqueous media on the structure and activity of lipases were systematically summarized. Moreover, various strategies that can be used in the engineering of lipases with high solvent tolerance have been highlighted and deeply discussed in this comprehensive review.

## 2. Overview of Lipases

Lipases, or acylglycerol hydrolases, are widely derived from animals, plants, insects, and microorganisms. As a member of the serine hydrolase family, lipases can catalyze numerous reactions such as hydrolysis, esterification, transesterification, and aminolysis. The high catalytic activity, enantioselectivity, and substrate specificity of lipases have made them the most widely used industrial enzymes [49].

**Figure 1:**
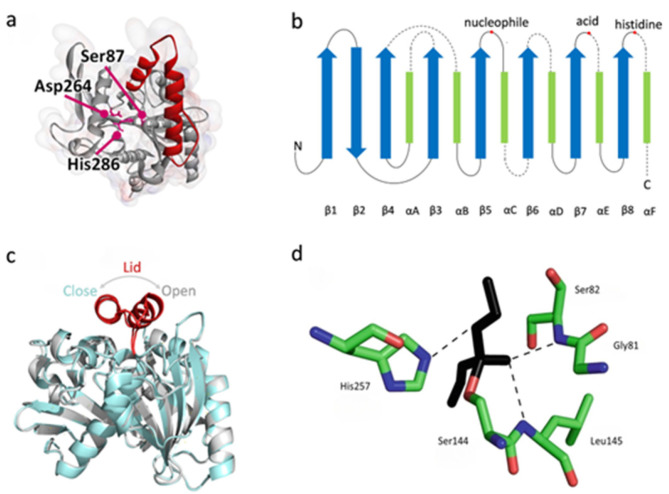
Structural features of lipases. (**a**) Three-dimensional structure of *Burkholderia cepacia* lipase (BCL; PDB code: 3LIP) with active site (Ser87, Asp264, and His 286, in magenta) and lid (118–159, in red). Reproduced with permission from refs [53]. Copyright 2020 American Institute of Chemical Engineers. (**b**) The canonical α/β-hydrolase fold. (**c**) The crystallographic structure of *Rhizomucor miehei* lipase with its α/β-hydrolase fold in gray exemplifies the lid in its open conformation (PDB code: 4TGL) and its α/β-hydrolase fold in cyan exemplifies the lid in its closed conformation (PDB code: 3TGL). The lids in both structures are in red. Reproduced with permission from refs [54]. Copyright 2020 by the authors. Licensee MDPI, Basel, Switzerland. (**d**) The oxyanion hole in *Rhizomucor miehei* lipase (PDB code: 4TGL). Diethyl phosphonate is stabilized by hydrogen bonds with Ser82 and Leu145. Substrate is shown in black and hydrogen bonds are schematized by dotted lines. Reproduced with permission from refs [52]. Copyright 2018 Springer Science + Business Media, LLC, part of Springer Nature.

Lipases vary tremendously in their primary structure, with the only commonality being that they all have a pentapeptide sequence of Gly-X-Ser-X-Gly (in a few cases, the glycine is replaced by other small residues) [50]. In terms of secondary and tertiary structures, lipases have an α/β-hydrolase fold, a catalytic triad consisting of serine, histidine, and glutamate or aspartate, a lid involving “interfacial activation” (in a few cases, some lipases do not have typical lid structure such as *Candida antarctica* Lipase B), and an oxyanion hole which is important for stabilizing the intermediate [51] (Figure 1). In addition, disulfide bonds are widely present in lipases and play an important role in enzymatic stability and catalytic activity [52].

The catalytic processes of lipases generally begin with the opening of the lid and the exposure of the active site. The hydrophobic substrate diffuses to the active site, and then with the help of the catalytic triad, it goes through the intermediate complexes of the enzyme tetrahedron, acylase, and acyl-enzyme tetrahedron. Finally, the whole catalytic process ends up with the deacylation of the lipases [55]. Such a catalytic mechanism is based on its structural characteristics. Compared with other serine hydrolases, lipases have a unique “interfacial activation mechanism” due to their special lid structure, i.e., the lid tends to be closed in the aqueous or oil phase, and it tends to be open at the oil–water interface. The hydrophobic part of the lid helps lipophilic substrates bind to the active site, so the open state of the lid is also known as the active conformation [56]. In addition, the oxyanion hole is also an important component that affects the catalytic efficiency of lipases. The oxyanion hole of most lipases consists of two residues with the first one located between the β3 chain and the αA helix and the second one located in the pentapeptide motif at the C-terminal end of serine catalytic residues. These residues are usually amide-structured or positively charged, stabilizing the catalytic intermediates by hydrogen bonding with the negative oxygen carbonyl group of the catalytic intermediates [54,57,58].

## 3. Effect of Different Solvents on Stability and Activity of Lipases

### 3.1. Effect of Organic Solvents on Stability and Activity of Lipases

Lipases frequently exhibit decreased activity or even become inactive in polar organic solvents [59,60,61], which is related to various factors such as water stripping and hydrogen-bonding interactions. For instance, Cui et al. [62] proposed that polar solvents like DMSO can remove water from the surface of *Bacillus subtilis* lipase A (BSLA), establishing diverse interactions such as hydrogen bonding and van der Waals forces, which ultimately disrupt the secondary structure of the lipases, leading to a decrease in its activity. Similarly, in the case of the immobilized lipase Lipozyme TLIM in a composite solvent comprising the hydrophilic solvent N, N-dimethylformamide (DMF) and the hydrophobic solvent tert-amyl alcohol, a significant concentration of DMF markedly inhibited the enzyme’s activity, which can be attributed to the potent polar nature of DMF, which strips water from the enzyme’s surface, resulting in a reduction or complete loss of its catalytic activity [63].

In addition, the structure of lipases exhibits greater flexibility in polar organic solvents compared to nonpolar ones, which presents a double-edged sword for lipase-catalyzed reactions. At a low temperature or room temperature, where atomic fluctuations are naturally subdued, this flexibility can be advantageous. For instance, the relative activity of *Bacillus thermocatenulatus* lipase 2 (BTL2) was notably higher in 10–70% (*v*/*v*) 2-propanol and ethanol solutions than in toluene and cyclohexane solutions of equivalent concentrations at 25 °C. However, as the temperature rises to 60 °C, no activity was observed in ethanol solutions of high concentrations (50% and 70%), whereas lipase activity in cyclohexane solutions of corresponding concentrations increased by more than twofold. This discernible difference can be attributed to the excessive flexibility of the enzyme in polar organic solvents at higher temperatures, which may lead to solvent penetration, thereby inducing protein unfolding. In contrast, lipases in nonpolar solvents exhibit enhanced thermal stability, likely due to their higher rigidity [64]. Moreover, some other studies also suggested that excess polar or nonpolar solvents are detrimental to the activity and stability of lipases, which can be attributed to the fact that relative structural flexibility plays a key role in the activity of lipases; the excess rigidity of the structure may not be beneficial to the binding of substrates and the excess flexibility may lead to the denaturation of the secondary structure of lipases.

Lipases in nonpolar organic solvents tend to be more structurally rigid and stable than in polar solvents. Sadaf et al. [65] found that lipase from *Penicillium chrysogenum* showed a dramatic increase in helical content in the n-hexane system, which suggests that the structure of lipases in n-hexane is more stable. Another study [66] demonstrated that *Burkholderia cepacia* lipase (BCL) showed lower root-mean-square deviation (RMSF) in hexane than in methanol, indicating enhanced structural rigidity in hexane. However, enhanced stability or rigidity is not always favorable to the catalytic activity of the enzyme. On the one hand, this effect may allow the lipases to remain active or even enhance their activity in organic solvents. Sadaf et al. [65] incubated purified lipases in different polar and nonpolar organic solvents at a concentration of 50% (*v*/*v*) for up to 72 h and found that the activity of lipases was enhanced 1.2-fold and 1.3-fold in n-hexane and cyclohexane, respectively. On the other hand, excess rigidity can also result in a decrease in lipase activity. This reduction may result from the constrained movement within lipase’s secondary structure. In the study of Shehata et al. [64], it was observed that when the toluene concentration exceeded 50% (*v*/*v*), the activity of BTL2 significantly decreased, likely due to the increased structural rigidity of the lipases.

In contrast to polar solvents, it has been demonstrated that lipases in nonpolar organic solvents adopt a more compact structure and may even form new salt bridges. Shehata et al. [64] found that the lipases maintain a more condensed structure in nonpolar solvents, regardless of the lid conformation (open or closed), as analyzed by the radius of gyration of BTL2 at 310 K. Similarly, Yenenler et al. [67] found that the distances between carbon atoms in the catalytic residues of BTL2 in toluene were smaller than those in water at 310 K and higher temperatures (450 K). This suggests that the hydrophobicity of toluene leads to a tighter stacking of the catalytic triplet and increased structural rigidity of the active site. Consequently, this heightened rigidity may hinder the induced-fit effect, preventing the substrate from binding to the active site properly. In addition, they found that BTL2 showed salt bridges in toluene that were not present in water and toluene–water mixtures (D8:R104, E134:K85, E128:R107, and E306:R204), which may also be one of the main reasons for the increased structural rigidity of the lipases.

### 3.2. Effect of Ionic Liquids on Stability and Activity of Lipases

**Figure 2:**
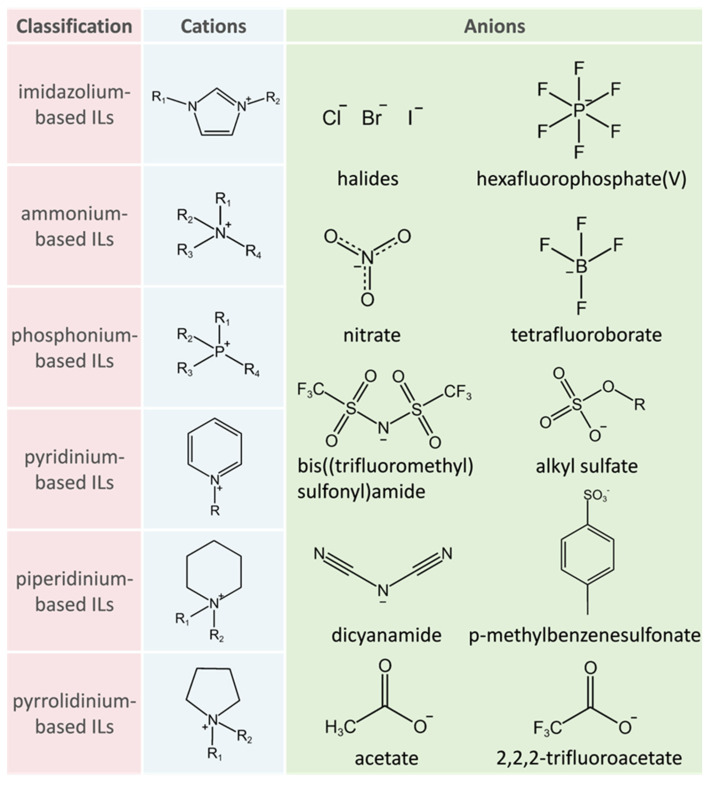
Some cations and anions of commonly used ILs.

ILs are a series of salts that are liquid at temperatures below 100 °C and are composed of organic cations and inorganic anions. The structures of several commonly employed cations and anions are illustrated in Figure 2. Because of their advantages such as low vapor pressure and nonflammability, ILs are considered to be highly promising green solvents and are expected to replace toxic, flammable, and volatile organic solvents [68]. ILs also exhibit good biocompatibility. In amine-based ILs, the proton transfer from acid to base results in the formation of a hydrogen-bonding network resembling that in aqueous solutions. This network imparts a buffering effect, allowing for the high stability of proteins over a broad pH range [69]. Furthermore, ILs are designable. Properties such as polarity, hydrogen-bonding network, and hydrophobicity, which are important factors affecting the stability and activity of lipases, can be designed and tailored by simply changing the composition of anions and cations as well as their ratios [70,71].

The hydrophobicity of ILs has been demonstrated to be primarily determined by the anion, for example, ILs containing tetrafluoroborate ([BF_4_]^−^) are likely hydrophilic, while ILs containing hexafluorophosphate ([PF_6_]^−^) tend to be more hydrophobic [71]. Additionally, the hydrophobicity of ILs will also increase with the length of the alkyl chain of the cation. In most cases, hydrophobic ILs will lead to increased enzyme activity, whereas hydrophilic ILs tend to decrease enzyme activity due to the fact that the hydrophilic ILs may strip water, which is crucial in maintaining activity, from the enzyme surface [70]. It has been observed by Ventura et al. [72] that as the concentration of several hydrophilic 1-butyl-3-methylimidazolium cation ([C_4_mim]^+^)-based ILs was increased, the activity of *Candida antarctica* lipase B (CALB) invariably showed a decreasing trend, which could be attributed to the fact that the hydrophilic ILs competed for the bounded water on the surface of the lipase, making the enzyme difficult to maintain its structural flexibility. In some cases, lipase activity in hydrophilic ILs may increase owing to a better solubility of polar substrates. For example, Lin et al. [73] found that for ILs with the same cation, the more hydrophilic the anion, the higher the conversion rate of the enzymatic reaction to synthesize lauroyl glucose ester, possibly because hydrogen-bonding interactions between the polyhydroxy groups of glucose and the hydrophilic anion facilitated glucose solubilization. Moreover, some studies have indicated that when the hydrophobic alkyl chains of IL cations are longer, the anticipated increase in solvent hydrophobicity does not positively impact enzyme activity as expected. Instead, it has the potential to adversely affect enzyme activity. For instance, Kim et al. [74] found that in the presence of several imidazolium-based ILs, CALB in 1-octyl-3-methylimidazolium trifluoromethanesulfonate ([Omim][TfO]) whose cation has the longest hydrophobic alkyl chain, showed significantly decreasing activity and reduced structural stability. It is probably because its longer alkyl chain can easily form hydrophobic interactions with L278, which may destroy the hydrogen bonding between L278 and A282, leading to the destruction of the α-10 helix structure, the increase in the volume of the catalytic cavity, and the exposure of the catalytic triplet to the solvent.

In addition to hydrophobicity, certain ILs can affect enzyme activity and stability through hydrogen bonding. Generally, ILs containing anions with a greater ability to form hydrogen bonds (e.g., Cl^−^, Br^−^, HCOO^−^) tend to result in a greater loss of enzyme activity than ILs containing perfluoroalkylsulfonate ([Tf_2_N]^−^), hexafluorophosphate ([PF_6_]^−^), and tetrafluoroborate ([BF_4_]^−^) [75], which is probably because the former interacts with the lipase molecules through hydrogen bonding, leading to the dissociation of the hydrogen bonds that maintain the enzyme structure [76]. Klähn et al. [77] showed that anions with compact size and strong charge polarization in ILs form strong hydrogen-bonding interactions with the enzyme, which results in the loss of the enzyme molecule’s denseness, the unraveling of the α-helix, and a decrease in stability.

### 3.3. Effect of Deep Eutectic Solvents on Stability and Activity of Lipases

**Figure 3:**
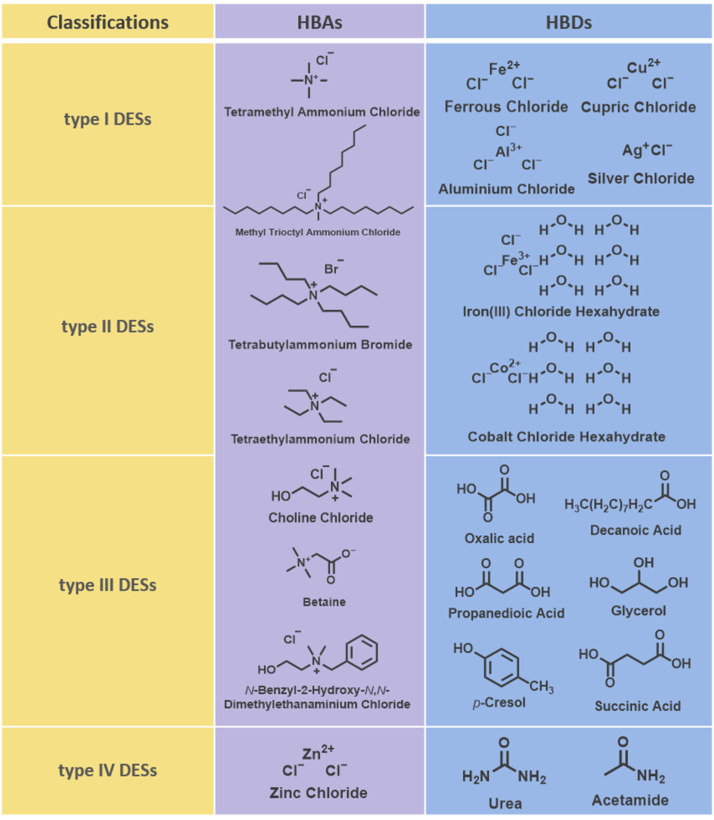
Classification of DESs and structures of some HBAs and HBDs.

DESs are a new type of eutectic-mixed solvent formed by the complexation of a hydrogen bond acceptor (HBA) and hydrogen bond donor (HBD). Based on the nature of HBDs, DESs can be categorized into four types: type I DESs consisting of quaternary ammonium salts and metal chlorides, type II DESs consisting of quaternary ammonium salts and metal chlorides hydrate, type III DESs consisting of quaternary ammonium salts and a number of different HBDs (e.g., amides, carboxylic acids, or polyols), and type IV DESs consisting of transition metals and HBDs (e.g., urea, acetamides, etc.) [78,79]. The classification of DESs and structures of some HBAs and HBDs are shown in Figure 3. DESs are considered to be a new green alternative solvent to ILs, and their non-toxic, biodegradable, easy-to-develop, and inexpensive characteristics make them valuable for a wide range of research and applications. In most DESs, enzymes can maintain a state of high activity and stability, which is more favorable to enzymatic synthesis reactions [78].

Studies have shown that some DESs have a stabilizing effect on lipase activity [80], which may be due to the fact that the addition of DESs attenuates the inhibitory effect of other substances in the reaction system on lipase activity. For example, He et al. [81] found that the addition of a choline chloride and glucose-based DES to a lipase-catalyzed biodiesel production system resulted in a significant increase in biodiesel yield, which may be attributed to the fact that the addition of DES avoids the inhibition of lipase activity via excessively high concentrations of methanol.

In addition, DESs can activate lipases or enhance their stability by forming suitable hydrogen bonds. Nian et al. [82] found that DESs can activate CALB by forming hydrogen bonds with the reaction substrate, which may be related to the fact that hydrogen-bonding interactions increase the nucleophilicity of the substrate, leading to an enhanced electron-withdrawing capacity of the substrate. At the same time, the amino acid residues on the surface of CALB can form hydrogen bonds with DESs to stabilize the secondary structure of the enzyme and enhance the stability of lipases. Álvarez et al. [83] concluded that the activity level of CALB in glycol-based DESs was higher than that in other DESs, which may be due to the fact that the hydrogen bonds formed between these types of solvents and CALB are weaker and do not result in a structural change that causes the loss of lipase activity. Coincidentally, Hollenbach et al. [84] found that the ester exchange activity of lipases in urea-based DESs was significantly higher than that of glucose-based DESs due to similar reasons. Through circular dichroism, Ma et al. [85] found that when DESs were used as a reactive medium, the proportion of β-turn in the structure of *Candida rugosa* lipase (CRL) was significantly increased, which in turn stabilized the secondary structure of CRL. Analysis of molecular dynamics (MD) simulations demonstrated that stabilizing hydrogen bonds were formed between DESs and CRL, which could significantly increase the affinity between CRL and its substrates and enhance the catalytic activity of CRL.

It is important to note that DESs do not consistently enhance the activity and stability of lipases. For instance, certain DESs can diminish lipase activity by forming destructive enzyme–substrate complexes or intermediate complexes [84]. However, in a broader context, as a novel category of eutectic-mixed solvents, DESs are considered environmentally friendly and green, suggesting a promising future in the field of enzymatic reactions. The effect of different deep eutectic solvent systems on the activity of some lipases and some examples of lipase-catalyzed reactions in a DES system are shown in Table 2 and Table 3, respectively.

### 3.4. Effects of Different Non-Aqueous Solvents on Selectivity of Lipases

One of the significant advantages of lipases is their good reaction selectivity, which has been proven to be influenced by the solvent in addition to factors such as temperature.

Polar organic solvents sometimes have the effect of increasing the selectivity of lipases. Wang et al. [95] catalyzed the synthesis of 1,3-diolein from oleic acid and glycerol using Lipozyme TL IM and found that the 1,3-position selectivity of the reaction was better in the relatively hydrophilic solvents, tert-butanol and tert-pentanol, compared to the hydrophobic solvents. MD simulations showed that the lid conformation of the lipases in hydrophilic solvents was more flexible and more easily activated, leading to a higher selectivity of lipases. In addition, elevating the content of polar organic solvents can also modulate the selectivity of lipase-catalyzed reactions. For example, Ehlert et al. [96] used DMSO miscible with water as a solvent in the enzymatic synthesis of asymmetric biphenyl esters. When the volume fraction of DMSO was elevated from 10% to 40%, the selectivities of the four lipases used to catalyze the reaction, respectively, were increased. However, the biocatalytic reaction rate was reduced, which may be related to the reduced stability of the lipases. It has been demonstrated that the addition of appropriate amounts of polar solvents to nonpolar organic solvents has a similar effect on the selectivity, but the amount of these solvents needs to be carefully controlled; otherwise, it may lead to a decrease in the enzyme activity.

As for ILs, lipases appear to maintain a relatively high selectivity in aqueous buffers or organic solvents to which ILs have been added. In the case of the hydrolysis of butyl 2-(4-chlorophenoxy)propionate by CRL, for example, the addition of hydrophobic ILs maintained 99% optical purity while obtaining higher yields compared to the addition of DMSO to the buffer [97]. Kołodziejska et al. [98] have also observed higher selectivity in the ester exchange reaction catalyzed by BCL in a mixture of 1-butyl-3-methylimidazolium hexafluorophosphate ([Bmim][PF_6_])/tert-butyl methyl ether (1:1, *v*/*v*); the researchers believed that the increased selectivity may be related to the hydrophobicity and high polarity of the ILs. In addition, for some reactions, temperature changes can invert the enantioselectivity of the lipases when ILs are used as solvents. Bustos-Jaimes et al. [99] used *Bacillus pumilus* lipase A to catalyze the synthesis of 1-phenylethanol hexyl ester in 1-ethyl-3-methylimidazolium methylsulfate ([Emim][EtSO_4_]) or 1-ethyl-3-methylimidazolium methanesulfonate ([Emim][CH_3_SO_3_]). Through a change in temperature alone, products achieved enantioselective inversion with either (R)- or (S)-enantiomers. However, this reversal is not present in hydrolysis reactions or in synthetic reactions using hexane as a solvent.

The synthesis of chiral compounds using DESs is feasible and promising as well. Fredes et al. [100] reported a Novozym 435-catalyzed asymmetric hydrolysis of dimethyl-3-phenylglutarate and they found that the selectivity of Novozym 435 was increased by 16% using choline chloride (ChCl): urea/phosphoric acid buffer (1:1, *v*/*v*) as the reaction medium compared to using 100% buffer as the solvent. However, not all DESs were effective in improving lipase selectivity. In the Novozym 435-catalyzed synthesis of 1,3-diacylglycerol, the ChCl-based DES system showed higher selectivity for 1,3-diacylglycerol, which may be attributed to the strong hydrogen-bonding network of this type of DES which may affect the environment of the enzyme’s active center and the water activity of the system. However, betaine-based DESs had no effect on enzyme selectivity although betaine is a quaternary ammonium salt just like ChCl [101].

### 3.5. Recovery of Lipases and Separation of Products in Different Non-Aqueous Solvents

After the lipase-catalyzed reaction in organic solvents, the problem of product separation and enzyme purification is inevitable. By taking advantage of the volatility of most organic solvents and the density difference of different organic solvents, it is relatively easy to achieve product separation with the help of distillation and liquid–liquid extraction. For the purification and recovery of lipases, although some classical methods such as ultrafiltration, precipitation, liquid–liquid partitioning, and chromatography are available, these methods have the drawbacks of easily inactivating the enzyme as well as consuming too much time, energy, and chemicals. Therefore, sustainable lipase purification strategies are yet to be developed. Currently, aqueous two-phase systems (ATPSs) have been demonstrated to be an alternative method of separating and purifying biomolecules with advantages such as low cost and less use of chemicals. For example, lipases can be separated from the fermentation broth using aqueous two-phase flotation, and the separation efficiency is maintained at more than 70% [102]. However, one of the reasons why this method has not yet been widely and commercially used may be that the partitioning mechanism involved in ATPSs is complex and needs to be further explored.

As for ILs, although lipase catalysis in ILs has numerous advantages, it has an obvious disadvantage—ILs are expensive, relative to organic solvents. Thus, ILs’ recovery and reuse are essential to expand their use from laboratory-scale to large-scale industrial applications. It has been mentioned earlier that the properties of ILs can be modulated by anions and cations, so the recovery methods are not uniform for ILs.

Regardless of the hydrophilicity or hydrophobicity of ILs, if the ILs contain low-boiling compounds, the low vapor pressure of ILs can be utilized to recover ILs by distilling out the low-boiling compounds. For biphasic systems containing hydrophobic ILs, recovery is relatively easy, as is evident in studies of biodiesel production. Biodiesel is a clean, biodegradable fuel that is intrinsically a mixture of fatty acid methyl esters (FAMEs), produced from a variety of crude oil materials (e.g., vegetable oils, animal fats, and waste oils) by transesterification reactions of triglycerides with methanol or ethanol. Lipase-catalyzed transesterification is very promising due to mild reaction conditions and low energy consumption. In addition, there is a growing trend towards the use of ILs in biodiesel synthesis probably because they can reduce enzyme inactivation caused by methanol and it is easy to recycle them [103]. A biphasic system is formed during biodiesel production, so simple centrifugation and decantation can separate the product from hydrophobic ILs, but this method has a narrow range of applications because it is based on the formation of biphasic states due to density differences and immiscibility. Sometimes, the solubility and density differences between ILs and certain organic solvents can be exploited, e.g., by adding cyclohexane to extract the product, after which the remaining IL–enzyme system can be recycled several times [104]. However, for the reaction of hydrophilic IL systems, the recovery of ILs is much more difficult and challenging, probably due to the impossibility of extracting the water-soluble substances by liquid–liquid extraction using water or more hydrophilic solvents. Therefore, new methods such as supercritical CO_2_-recovering ILs need to be developed.

In biocatalytic systems with DESs, lipases (especially immobilized lipases) can be removed relatively easily by precipitation, centrifugation, or filtration, but the separation of the downstream target product from the DESs remains a great challenge, which is related to the low vapor pressure properties of DESs that distinguish them from volatile organic solvents, and separation strategies need to be determined on a case-by-case basis. For example, Pätzold et al. [105] used two DES compounds -(−)-menthol (in excess) with dodecanoic acid as both substrate and solvent in a lipase-catalyzed esterification reaction. Since (−)-menthol has a lower boiling point than both dodecanoic acid and the ester, it can ultimately be recovered by distillation under reduced pressure. The conversion of dodecanoic acid when the recovered (−)-menthol was reutilized for esterification was almost close to 100%. Also in the case where the two components of DESs were used as substrates, Lozano et al. [106] added limonene to the reaction solution containing the unreacted panthenol and the product panthenol monolaurate (also containing a small amount of lauric acid), with the ester going into the limonene phase and the panthenol remaining in the other phase awaiting reuse.

However, for lipase-catalyzed reactions with DESs acting only as solvents, solvent–product separation may be more difficult because of the more complex compound fractions in the system. When DESs are involved in biocatalytic reactions as solvents only, anti-solvent addition, solid–liquid extraction, liquid–liquid extraction, short-range distillation, and methods based on density differences can be used to recover and recycle the DESs. For instance, Panić et al. [107] catalyzed the synthesis of (R)-1-phenylethanol using Novozyme 435 and selected ChCl: glycerol as the solvent. The product (R)-1-phenylethanol was separated by liquid–liquid extraction via adding ethyl acetate, and the DES and substrate were left in the other phase to be recovered and reused. In addition, when liquid *Yarrowia lipolytica* lipase 2 was used to catalyze the synthesis of biodiesel, a deep eutectic solvent based on ChCl and glucose was added. Ultimately, by utilizing the density and viscosity difference between oil and DESs, the DES’s reuse was achieved by centrifuging directly and taking the lower layer (similarly to ILs). When the DES was used for the second time, the biodiesel yield reached 99.6% [108].

It can be seen that most of the current methods still require large amounts of solvents (e.g., water, organic solvents) to aid in the separation, which often leads to high energy consumption, high cost, and is not green. Therefore, more environmentally friendly and efficient methods are yet to be explored and developed.

## 4. Protein Engineering for Improving the Solvent Tolerance of Lipases

**Figure 4:**
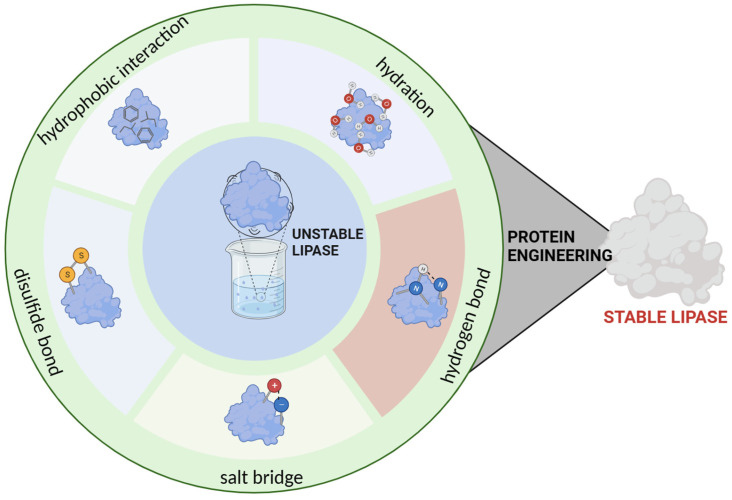
Some strategies of lipase engineering to improve their tolerance to solvents (The figure was prepared using the online tool, BioRender).

As opposed to traditional biocatalysis in aqueous solutions, biocatalysis in the non-aqueous phase has the advantages of altering the regioselectivity and enantioselectivity of the enzyme, avoiding bacterial contamination, and avoiding water-dependent side reactions [109,110]. However, lipases in non-aqueous phases are often adversely affected. Enzymes in organic solvents have lower catalytic activity compared to those in water [59], and certain anions and cations with long alkyl chains in ILs often lead to destabilization of the lipase structure [72]. Enzymes in DESs face challenges as well. For instance, organic acid-based DESs have been demonstrated to have adverse effects on enzymes possibly owing to an inappropriate pH environment [111]. These circumstances make it important to engineer lipases for better solvent tolerance. In previous studies, the most widely used strategies in the engineering of lipases solvent tolerance include the following: (1) hydration-based protein engineering, (2) reconstruction of the hydrogen-bonding network, (3) redesign of the salt bridge, (4) introduction of the disulfide bonds, and (5) adjustment of the hydrophobic interaction (Figure 4).

### 4.1. Hydration-Based Protein Engineering for Improving the Solvent Tolerance of Lipases

Water plays a crucial role in maintaining lipase activity because some bounded water on the enzyme surface can prevent the interaction with solvents and thus help maintain the conformation of enzymes [112]. It was noted that the degree of BSLA hydration is positively correlated with its tolerance in organic solvents and that penetration of polar organic solvents accompanied by stripping of water molecules results in the destruction of the hydration layer on the lipase’s surface, a decrease in the degree of hydration, and a corresponding decrease in the stability of the lipases [113]. Similar conclusions have been achieved by Pramanik et al. [114] in the IL system and Wang et al. [115] in the DES system. They found that compared to nonbeneficial variants and the wild type, the BSLA variants with increased solvent tolerance had a significantly higher number of water molecules on their surface.

Surface charge engineering is a commonly used method to improve the solvent tolerance of lipases by maintaining the hydration shell on the enzyme surface. Markel et al. [116] screened for BSLA variants with improved tolerance in water-miscible 1,4-dioxane (DOX), 2,2,2-trifluoroethanol (TFE), and DMSO after random mutagenesis, and found that the beneficial substitutions mainly involved the introduction of charged or polar amino acids. Some of the introduced charged residues formed new salt bridges, but more importantly, charged amino acids attracted water molecules, resulting in enhanced hydration and maintenance of the hydration layer on the enzyme surface. Moreover, some studies have focused on reducing organic solvent penetration through other methods. To improve the tolerance of BSLA in water–ethanol co-solvents, Min et al. [117] used computer tools to identify ethanol contact sites and then performed computer-simulated saturated mutagenesis. Finally, they found that the stability of the quadruple mutant combining S16G, A38G, A38T, and L108N was 1.8-fold higher than that of the wild type in 50% (*v*/*v*) ethanol, which was probably because the four mutated amino acids are no longer in contact with ethanol, which means ethanol penetration is reduced, and solvent–protein interactions are weakened. Similarly, Dror et al. [118] suggested that the Q185L variant of *Geobacillus stearothermophilus* lipase T6 tends to maintain a closed lid conformation that restricts the entry of methanol and excess substrate. In particular, a solvation-guided engineering strategy was formulated by Sheng et al. [119] to achieve BSLA resistance to DESs. They established a solvation score to comprehensively evaluate the solvation state of amino acids on the surface, taking both hydration and DES effects into consideration. Then, amino acids with low scores were substituted with amino acids with high scores. The final results showed that all four variants exhibited higher activity than the BSLA wild type at all the concentrations in the three DESs, proving the feasibility of the strategy.

### 4.2. Hydrogen Bond-Based Protein Engineering for Improving the Solvent Tolerance of Lipases

Hydrogen bonds play a key role in the activity and stability of lipases due to the maintenance of protein conformation and the binding of the substrate [120,121]. For instance, the resistance of the S194R variant of *Bacillus subtilis* lipase to several organic solvents was significantly improved due to the formation of several new hydrogen bonds [122]. Moreover, Kawata et al. [123] also suggested that the formation of five new hydrogen bonds of lipase LST-03 from *Pseudomonas aeruginos*a improved its tolerance in DMSO and various types of straight-chain alkanes. Additionally, the stable hydrogen-bonding network at the BSLA active site was proven to help maintain the enzyme’s tolerance to 15% (*v*/*v*) 1-butyl-3-methylimidazolium trifluoromethanesulfonate ([Bmim][TfO]) [124].

Schwaneberg et al. [125] suggested that the introduction of charged and polar amino acids may be beneficial to the solvent tolerance of lipases due to the formation of new hydrogen bonds with other residues or water molecules. Some other researchers pointed out that charged and polar substitutions at different positions have different effects. For instance, Frauenkron-Machedjou et al. [76] found that polar substitutions tended to yield more beneficial variants compared to charged substitutions at BSLA’s internal buried positions probably because it is difficult to find residues of corresponding charge to form salt bridges by introducing charged residues whereas introducing polar residues can stabilize the lipases by hydrogen bonding. However, in the exposed positions on the surface of the enzyme, the conclusion was reversed, i.e., charged substitutions tended to be more beneficial than polar substitutions.

Forming hydrogen bonds to stabilize lipases in solvents is not exclusive to charged and polar amino acids. For example, Dror et al. [126] replaced the charged residues on the surface with hydrophobic residues, improving the stability of lipase T6 from *Geobacillus stearothermophilus* in a mixed solvent of methanol–water. The visualized structure of the beneficial variant demonstrated new hydrogen bonds formed between the lipases and the structural water. Afterward, when the previously substituted charged residues were replaced with alanine, the new variant became unstable. The difference between the two sets of substitutions indicates that the stability of the variants after the substitution of hydrophobic residues does not arise from the elimination of surface charge or π interactions but from the formation of new hydrogen bonds on the lipase’s surface.

### 4.3. Salt Bridge-Based Protein Engineering for Improving the Solvent Tolerance of Lipases

Salt bridges are electrostatic interactions between two groups with opposite charges, which are formed between basic amino acids (lysine, arginine, histidine) and acidic amino acids (aspartate, glutamate) at a suitable distance [127]. Salt bridges influence the structure, function, and stability of proteins [128]. Many studies have identified newly formed salt bridges in lipase variants with improved organic solvent tolerance [122,123,129]. To investigate the contribution of salt bridges near the active site to the stability of *Proteus mirabilis* lipase (PML) in organic solvents, Dachuri et al. [130] substituted alanine for two arginine residues (R237 and R241) and one aspartate residue (D248) which formed salt bridges near the active site and then evaluated their efficiency in 10–40% (*v*/*v*) DMSO and 10–40% (*v*/*v*) methanol. The results showed that the double mutant (R237A/D248A) even lost its activity completely within 1 h in the 40% organic solvent, whereas the wild type had about 80% activity in both 40% (*v*/*v*) organic solvents. The dramatic decrease in activity may be due to the fact that the two pairs of salt bridges near the active sites play an important role in maintaining the correct catalytic conformation, and once they are removed, the structural stability of the enzyme is damaged.

However, salt bridges are not universally beneficial for lipase stability. For example, Cui et al. [131] suggested that the substitution of oppositely charged amino acids can disrupt the unfavorable salt bridges (bridges not present in a buffer but formed in organic solvents) and thus improve the solvent resistance of BSLA, which suggested that the breaking of unfavorable salt bridges may facilitate the restoration of the interaction network among surface residues, consequently enhancing the lipase’s solvent tolerance.

### 4.4. Disulfide Bond-Based Protein Engineering for Improving the Solvent Tolerance of Lipases

Disulfide bonds are formed by the oxidation of two non-adjacent cysteines in proteins [132], which have a significant role in the solvent resistance, thermal stability, and functional regulation of proteins [133,134]. A number of recent studies have improved the thermal stability of lipases by introducing disulfide bonds [135,136,137,138], which may be attributed to the rigidity of lipases conferred by the disulfide bonds. However, the effect of disulfide bonds on lipase stability in various solvents remains unclear. Ogino et al. [139] showed that the disulfide bond between C30 and C58 plays an important role in maintaining the stability of the PST-01 protease in organic solvents. In comparison, Pulido et al. [140] found that although the lipase LipA from *Pseudomonas aeruginosa* PSA01 with disulfide bonds was more thermally stable than the lipases born lacking the disulfide bond, the former had a shorter half-life and faster inactivation in organic solvents (methanol and ethanol).

The introduction of disulfide bonds after identifying the site with the help of computer tools is a viable way to improve lipase’s tolerance to organic solvents. For example, Gihaz et al. [141] used computational tools to select the amino acid pairs with the highest likelihood of spontaneous disulfide bond formation once mutated to cysteines, and the final six mutants showed a 1.4- to 3.5-fold increase in activity compared to the wild type after incubation for 1 h in 70% (*v*/*v*) methanol. Moreover, in order to increase the rigidity of the lipases, Hamdan et al. [142] introduced disulfide bonds to tighten the N- and C-termini of the protein. The final mutants, 2DC (S2C and A384C), showed a significantly higher relative residual activity than the wild type after incubation in 40% (*v*/*v*) methanol for 30 min.

### 4.5. Hydrophobic Interaction-Based Protein Engineering for Improving the Solvent Tolerance of Lipases

Hydrophobic residues in proteins (e.g., valine, leucine, isoleucine, phenylalanine, etc.) tend to be buried inside the protein molecule, and the hydrophobic interaction has always been considered the main force driving the folding of globular proteins and is closely related to the structural stability of proteins [143]. Typically, hydrophobic residues are situated within the protein molecule. However, during protein denaturation, these residues may become exposed on the surface and engage in hydrophobic interactions with other proteins, leading to protein aggregation and precipitation [144]. Therefore, it appears more practical to enhance the solvent tolerance of lipases by introducing hydrophobic residues internally rather than on the surface of the enzyme.

Based on the role of aromatic side chains in the folding and thermodynamic stability of proteins [145], Gihaz et al. [146] introduced a large number of aromatic residues into the solvent tunnels of the lipase from *Geobacillus stearothermophilus* T6 and obtained a variant (L184F/A187F/L360F) with enhanced stability in methanol, which may be due to the fact that it not only limited the penetration of polar organic solvents into the core of the enzyme but also helped form new π–π interactions. In contrast, introducing hydrophobic amino acids on the enzyme’s surface is a desperate approach. Monsef et al. [147] replaced the polar amino acids on the surface of a novel lipase from *Pseudomonas* sp. (GQ243724) with hydrophobic amino acids, resulting in beneficial variants with improved tolerance in several polar organic solvents (methanol, ethanol, n-propanol, DMF). However, they also suggested that the selection of mutation sites needs to be carefully considered. For instance, when N219 on the enzyme’s surface was substituted by leucine, the solvent resistance of the mutant was enhanced owing to the hydrophobic interactions between L219 and V218, V221, and Y223, but when S251 on the enzyme’s surface was replaced by leucine, the stability of the mutant in organic solvents was rather reduced because S251 may participate in the hydration layer formation.

The hydrophobic interaction between long alkyl chains of IL cations and the hydrophobic region near the lipase’s active site can lead to the instability of enzymes [77]. Taking advantage of this understanding, Nordwald et al. [148] mutated the hydrophobic residue (G158) near the active site of BSLA to the acidic amino acid (glutamate), resulting in a 2.5-fold increase in lipase’s tolerance to 50% (*v*/*v*) 1-butyl-3-methylimidazolium chloride ([Bmim][Cl]). The mutation G158E inhibited the hydrophobic interaction between the hydrophobic region near the active site and the IL and thus led to an increase in solvent tolerance of the lipases. In addition to the introduction of acidic amino acids, the introduction of basic amino acids in the vicinity of the active site had a similar effect [124]. Some examples of protein engineering for improving the solvent tolerance of lipases are summarized in Table 4.

## 5. Conclusions and Outlook

Lipases have garnered significant attention in biochemical synthesis methods due to their notable catalytic activity, enantioselectivity, substrate specificity, and large substrate scope. However, the structural characteristics of lipases make them exhibit different properties in different types of solvents. To this end, this review summarizes the methods to improve the tolerance of lipases in different non-aqueous solvents and some examples of engineering lipases in recent years. As the concept of green chemistry takes hold, engineering lipases to enhance lipase stability in DESs is rather imperative. However, only a few studies have been reported to improve lipase’s tolerance to DESs by protein engineering. To address the problem, a computer-aided rational design of lipases can be adopted, whereby the mutation sites are predicted by a computer and mutagenesis is simulated, and then MD simulations are used to compare the structural stability of the mutant with that of the wild type in the solvent, and finally, mutants with improved solvent tolerance are obtained by analyzing a variety of parameters. The method is expected to improve the stability of lipases in DESs. The review compiles and summarizes the current research status of lipase tolerance in different solvents and its engineering, which provides a basis for further engineering and application of lipases.

## Figures and Tables

**Table 1:** The applications of lipases in various fields.

Application Fields	Specific Aspects	References
Food	Flavorings	[10,11]
	Production of human milk fat substitutes	[12,13]
	Egg processing	[14,15]
	Bread processing	[16,17]
	Cocoa butter substitute processing	[18,19]
	Edible oil processing	[6,20]
	Cheese processing	[21,22]
	Meat and fish processing	[23]
	Vitamin production	[24,25]
Textile	Textile processing	[26,27]
Papermaking	De-inking of wastepaper	[28,29]
Leather	Leather degreasing	[30,31]
Pharmaceuticals	Chiral drug preparation	[32,33]
	Diagnostic tools	[34]
	Anti-obesity treatment	[35]
	Medical device composition	[36]
Cosmetics	Personal care products production	[37,38]
	Perfume production	[39]
Detergents	Detergent production	[40,41]
Bioenergy	Biodiesel production	[42,43]
Environment	Wastewater treatment	[44,45]
	Plastic degradation	[46,47]
Agriculture	Herbicide synthesis	[48]

**Table 2:** The effect of different deep eutectic solvent systems on the activity of some lipases.

DES System	Lipase	Substrate	Temperature	Time	Relative Enzyme Activity	References
Bet:Gly (1:3)	CALB	pNPB	40 °C	5 min	95.00%	[80]
ChCl:Fru:H_2_O (5:2:5)	CALB	pNPB	40 °C	5 min	92.00%	[80]
ChCl:U (1:2)	CALB	pNPB	40 °C	5 min	87.00%	[80]
Bet:Gly (1:2)	CALB	pNPP	59.85 °C	5 min	115.48%	[82]
Bet:Xyl (1:1)	CALB	pNPP	59.85 °C	5 min	91.69%	[82]
DecA:Lid (2:1)	CRL	pNPP	40 °C	10 min	81.02%	[85]
ChCl:EG (1:2) H_2_O/DES (% *v*/*v*) = 70/25	LipC12	pNPP	25 °C	2 min	1050.00%	[86]
ChCl:Gly (1:2) H_2_O/DES (% *v*/*v*) = 70/25	LipC12	pNPP	25 °C	2 min	1233.00%	[86]
ChCl:Sor (2:1) H_2_O/DES (% *v*/*v*) = 70/25	LipC12	pNPP	25 °C	2 min	1795.00%	[86]
Ala:NaOH (1:1)	PPL	pNPP	40 °C	15 min	162.50%	[87]

Bet: Betaine; Gly: Glycerol; ChCl: Choline chloride; Fru: Fructose; U: Urea; Xyl: Xylose; DecA: Decanoic acid; Lid: Lidocaine; EG: Ethylene glycol; Sor: Sorbitol; Ala: Alanine; CALB: *Candida antarctica* lipase B; CRL: *Candida rugosa* lipase; PPL: Lipases from the porcine pancreas; pNPB: p-nitrophenyl butyrate; pNPP: p-nitrophenyl palmitate.

**Table 3:** Some examples of lipase-catalyzed reactions in a DES system.

DES	Lipase	Substrates	Temperature	Time	Conversion Rate	Reference
ChCl:glycerol (1:2) H_2_O/DES (% *v*/*v*) = 10/90	Novozym 435	benzoic acid, glycerol	60 °C	24 h	90%	[88]
(−)-menthol:decanoic acid (65:35) H_2_O/DES (% *w*/*w*) = 10/90	CRL	decanoic acid, (−)-menthol	35 °C	7 d	83%	[89]
ChCl:urea (7:6) (*w*:*w*)	Novozym 435	methyl caffeate, glycerin	65 °C	2.5 h	96.46% (yield rate)	[90]
Decanoic acid:(−)-menthol (1:1)	immobilized lipase B from Candida antarctica	vinyl decanoate, glucose	50 °C	24 h	10.95% (yield rate)	[91]
ChCl:glycerol (1:2)	Lipozyme RM IM	high-acid rice bran oil, glycerol	50 °C	24 h	94.5%	[92]
ChCl:urea (1:2)	immobilized lipase B from Candida antarctica	α-benzylcyclopentanones	55 °C	3 d	exceed 90%	[93]
ChCl:ethylene glycol (1:2) H_2_O/DES (% *w*/*w*) = 5/95	immobilized lipase B from Candida antarctica	acetic anhydride, 1-butanol	25 °C	2 h	80% (yield rate)	[94]

**Table 4:** Recent examples of engineering lipases to improve their solvent tolerance.

Lipases	Solvent	Beneficial Mutants	Stabilizing Factors	References
*Proteus mirabilis* lipase	methanol	a variant with 13 mutation sites (Dieselzyme 4)	hydrogen bond, salt bridge	[129]
*Bacillus subtilis* lipase A	12% (*v*/*v*) 2,2,2-trifluoroethanol	I12R/M137H/N166E	hydration	[113]
*Bacillus subtilis* lipase A	water-miscible organic cosolvents (1,4-dioxane, 2,2,2-trifluoroethanol, dimethyl sulfoxide)	introduction of charged and polar amino acids	hydration	[116]
*Bacillus subtilis* lipase A	1,4-dioxane, 2,2,2-trifluoroethanol, dimethyl sulfoxide	introduction of charged and polar amino acids	hydrogen bond	[125]
lipase from *Bacillus subtilis*	acetonitrile, dimethyl sulfoxide, N, N-dimethylformamide	M134D/I157M/Y139C/K112D/R33G	hydrogen bond, salt bridge	[122]
*Geobacillus stearothermophilus* lipase T6	methanol	H86Y, Q185L, and A269T	hydrogen bond, hydration	[118]
lipase LST-03 from *Pseudomonas aeruginosa*	dimethyl sulfoxide, straight-chain alkanes	S155L, S164K, S194R, and D209N (dimethyl sulfoxide), G157R, S194R, and D209N (straight-chain alkanes)	hydrogen bond, salt bridge, and hydration	[123,149]
*Geobacillus stearothermophilus* lipase T6	water-methanol cosolvent	H86Y/A269T/R374W	hydrogen bond	[126]
*Bacillus subtilis* lipase A	water-ethanol co-solvent	S16G, A38G, A38T, and L108N	hydration	[117]
a novel lipase from *Pseudomonas* sp. (GQ243724)	methanol, ethanol, n-propanol, N, N-dimethylformamide	N219L, N219I	hydrophobic interaction	[147]
*Candida antarctica* lipase B	40% (*v*/*v*) ethanol	V139E/A92E	flexibility	[150]
*Geobacillus stearothermophilus* lipase T6	methanol	E251C/G332C	disulfide bond	[141]
*Geobacillus zalihae* T1 lipase	methanol	S2C/A384C	disulfide bond	[142]
*Geobacillus zalihae* T1 lipase	dimethyl sulfoxide, n-hexadecane	L188M, A190L, A190Y, L188M/A190L, and L188M/A190Y	hydrophobic interaction	[151]
*Candida antarctica* lipase B	water-methanol cosolvent	N97Q, N264Q, and D265E	hydrogen bond, hydration	[113]
*Bacillus subtilis* lipase A	water-miscible organic cosolvents (1,4-dioxane, 2,2,2-trifluoroethanol, dimethyl sulfoxide)	D34K, D34R, K112D, K112E, K112D, and D144K	hydration	[131]
*Bacillus subtilis* lipase A	[Bmim][Cl], [Bmim][Br], [Bmim][I], [Bmim][TfO]	introduction of charged and polar amino acids	hydrogen bond, hydration	[76]
*Bacillus subtilis* lipase A	[Bmim][Cl]	G158E/K44E/R57E/Y49E	ionic interaction	[148]
*Bacillus subtilis* lipase A	ChCl: acetamide, ChCl: ethylene glycol, tetrabutylphosphonium bromide: ethylene glycol	T66H/G67D, K88E/N89K, M137D/N138D, M137D/N138H, Y161D/S162E/S163E	hydration	[115]
*Bacillus subtilis* lipase A	ChCl: acetamide, ChCl: ethylene glycol, tetrabutylphosphonium bromide: ethylene glycol	D64H/R107L/E171Y, D64H/R142L/E171Y	hydration	[119]

[Bmim][Cl]: 1-butyl-3-methylimidazolium chloride; [Bmim][Br]: 1-butyl-3-methylimidazolium bromide; [Bmim][I]: 1-butyl-3-methylimidazolium iodide; [Bmim][TfO]: 1-butyl-3-methylimidazolium trifluoromethanesulfonate; ChCl: choline chloride.
